# Supplementary material for: Structural genomic variation and migratory behavior in a wild songbird
Source: Evol Lett. 2023 Oct 7;7(6):401–12. doi: 10.1093/evlett/qrad040 (PMC10693001; doi:10.1093/evlett/qrad040)
Supplement: qrad040_suppl_Supplementary_Tables_S1_Figures_S1-S4 [file qrad040_suppl_supplementary_tables_s1_figures_s1-s4.pdf]

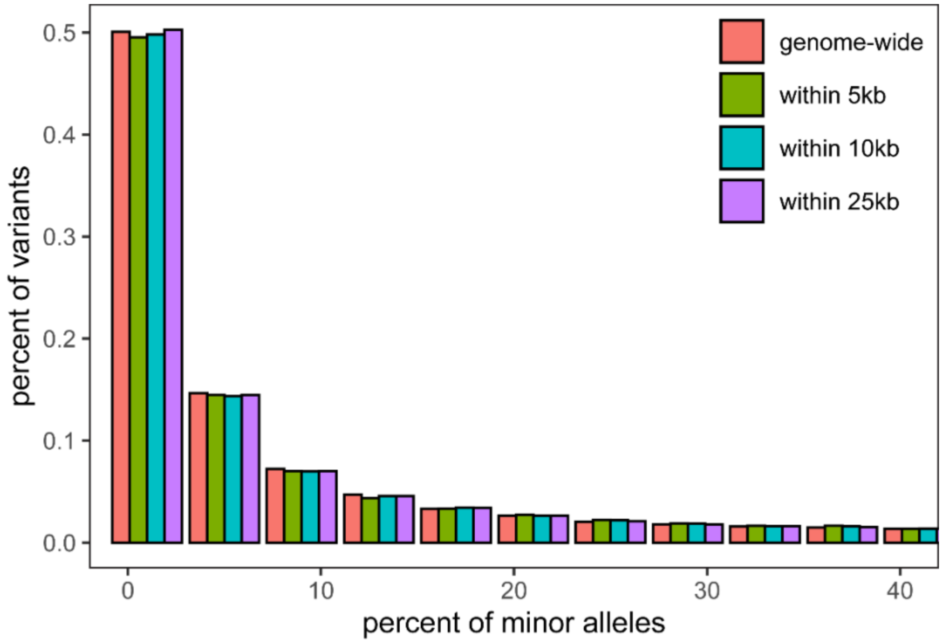

Fig. S1. Allele frequency spectrums (AFS) for SNPs within specific distances (bins) of SVs.

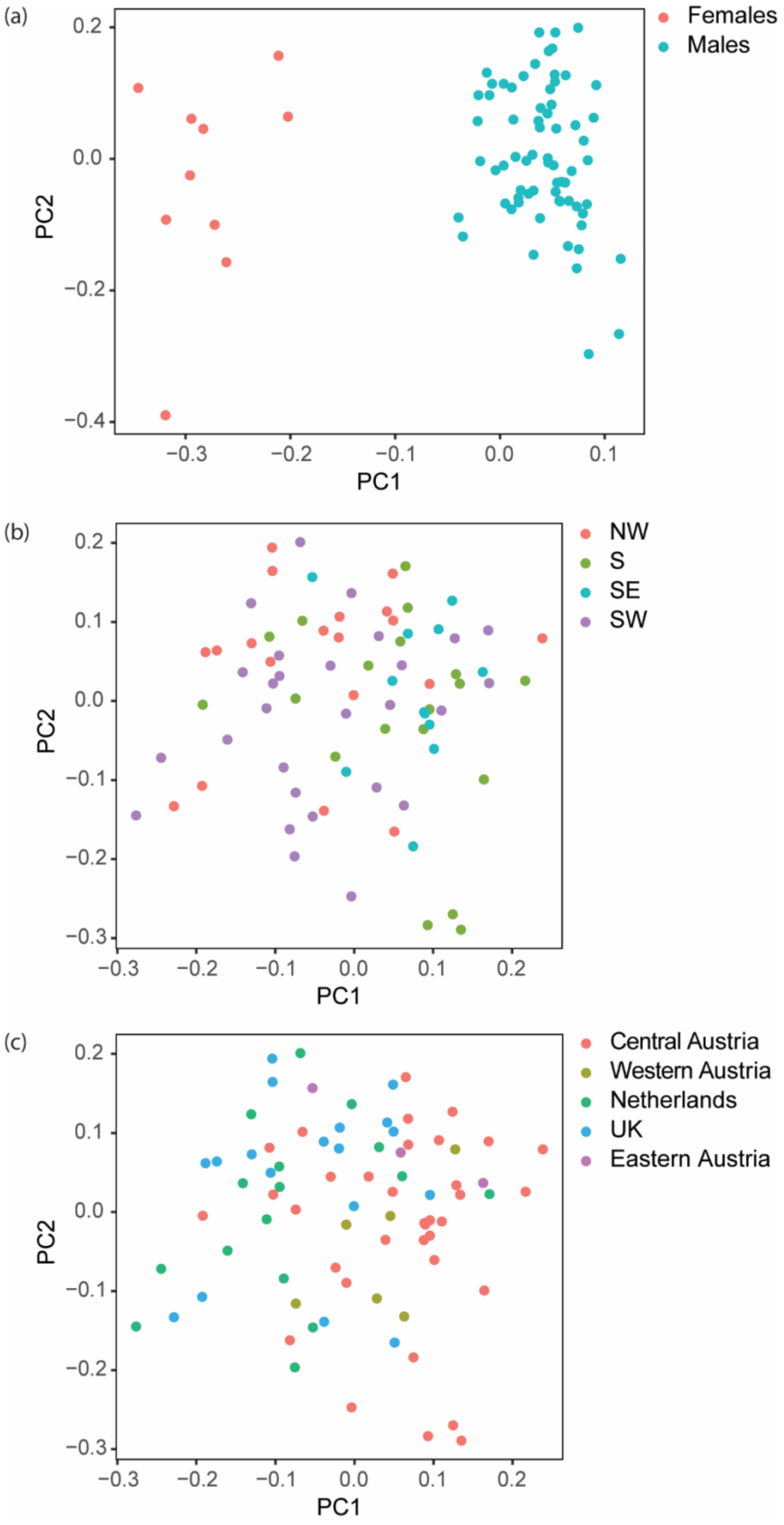
(a)
Females
Males
PC2
PC1
0.2
0.0
−0.2
−0.4
−0.3
−0.2
−0.1
0.0
0.1
(b)
NW
S
SE
SW
PC2
PC1
0.2
0.1
0.0
−0.1
−0.2
−0.3
−0.3
−0.2
−0.1
0.0
0.1
0.2
(c)
Central Austria
Western Austria
Netherlands
UK
Eastern Austria
PC2
PC1
0.2
0.1
0.0
−0.1
−0.2
−0.3
−0.3
−0.2
−0.1
0.0
0.1
0.2

Figure S2. Results from PCA analysis. (a) Shows results using structural variants from all chromosomes and distinguishes between the sexes. (b and c) Show results using autosomal structural variants and highlighting birds by migratory orientation (n = 19 NW, 28 SW, 12 SE and 20 S; Table S1) and deployment region, respectively.

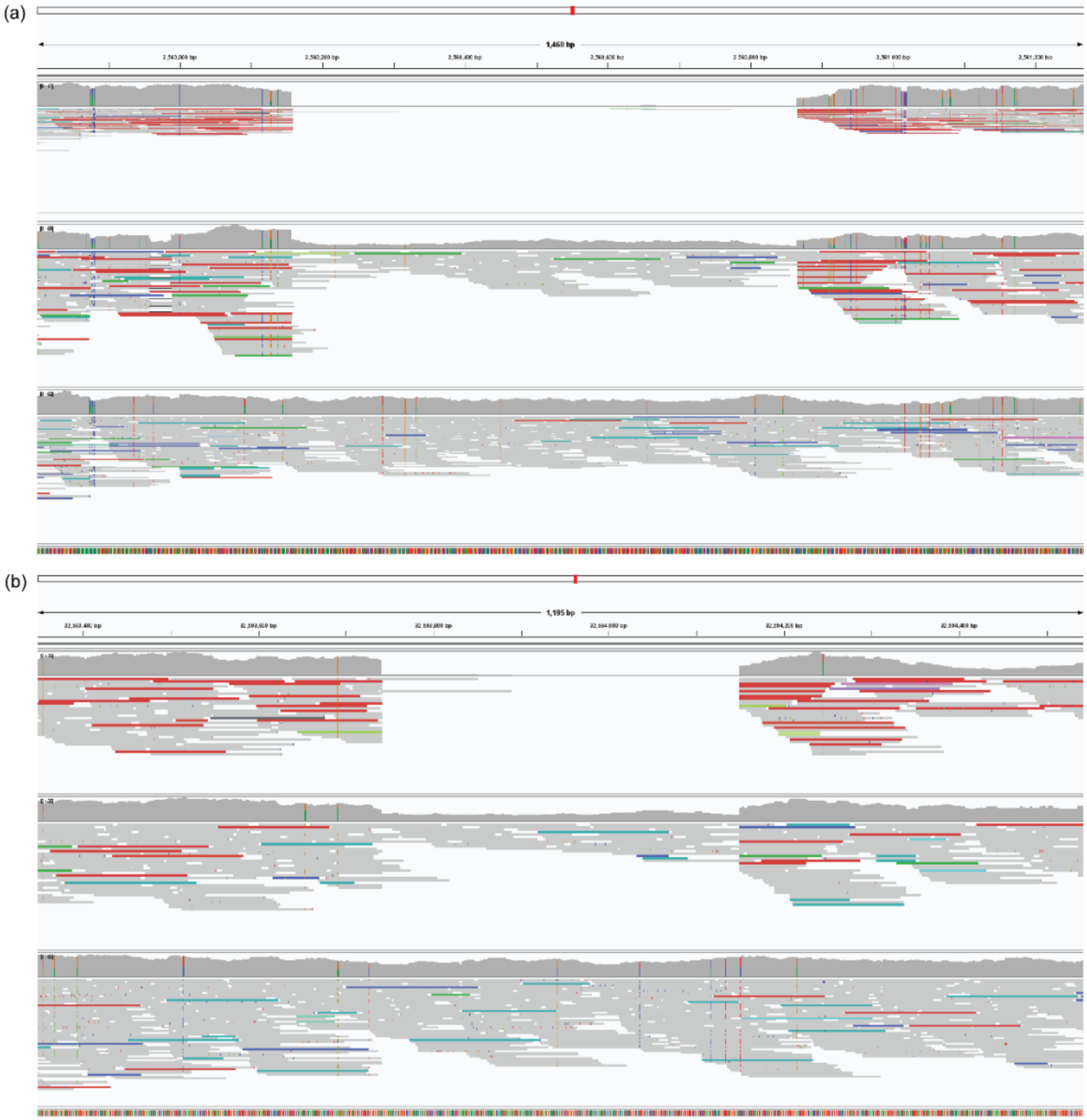

Fig. S3 Visualization of SVs using IGV. Examples of all three genotypes (0/0 top panel, 0/1 middle panel and 1/1 bottom panel) for two variants are shown, including the deletion on chromosome 27 that shows elevated PBS and an association with migratory direction (b).

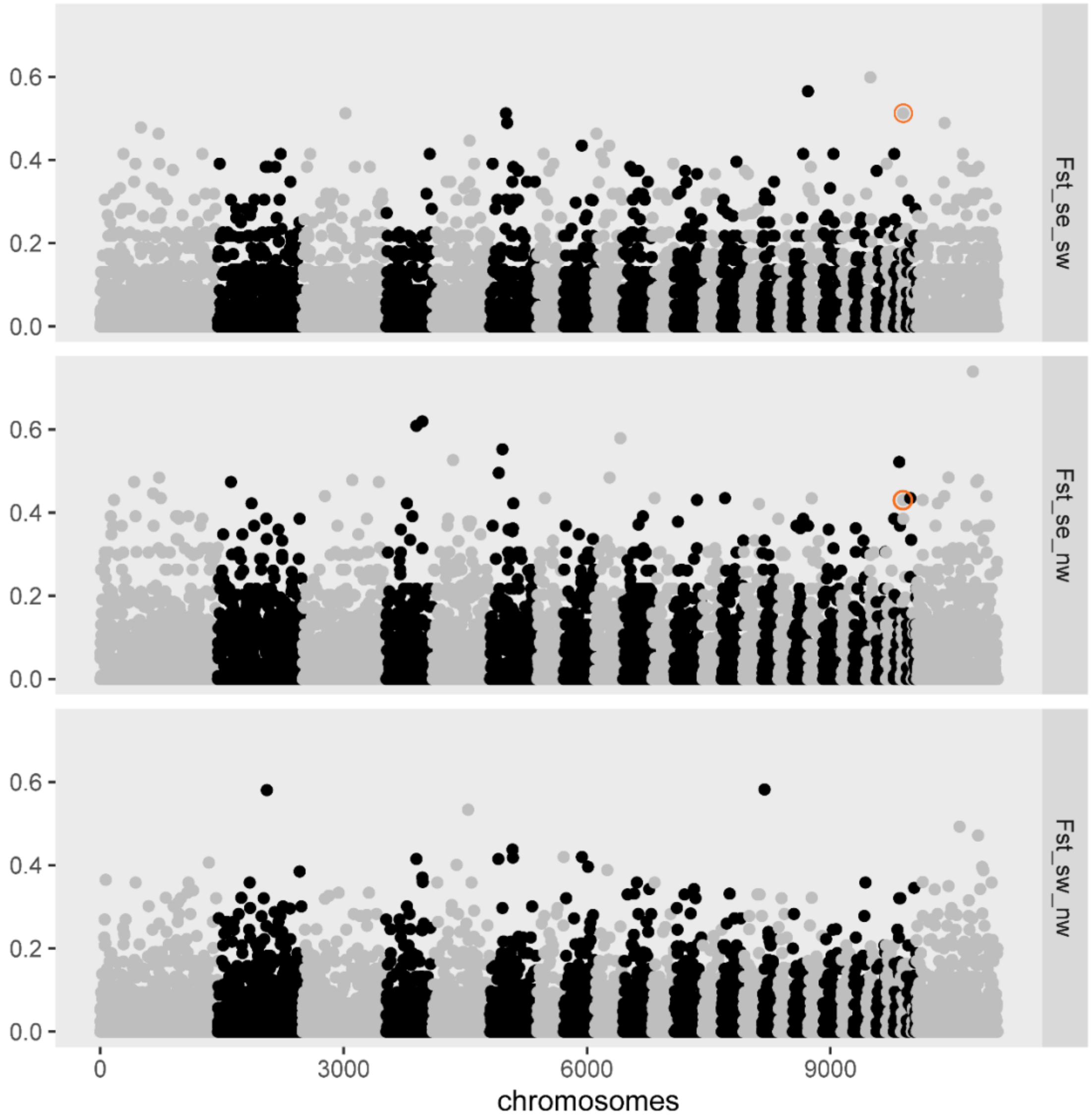

Fig S4. Estimates of FST between populations that migrated along NW, SW and SE routes from their breeding grounds. SV circled in orange was also identified in our genome-wide association analyses and is highlighted in Figure 3 which presented estimates of PBS derived from these estimates of FST.

Table S1. Information on samples used in study, including sex and location data along with information on sequencing conducted, subsequent assemblies and SVs called. Data on migratory phenotypes was published in Delmore et al. 2020 Proceedings of the Royal Society B, 287:20201339.

id	sex	deploy_ season	deploy_ region	lon_ summer	lat_ summer	winter_ region	mean_molecule_ length_bp	#_reads_ x1000000	mean_ depth	contig_ n50_kb	scaffold_ n50_mb	assembly_ size_gb	svim_ svs	mumco_ svs	lr_ svs
T012827	1	summer	Austria	9.66	47.49	SW	30527.98	533.68	54.07	110.12	8.36	1.00	36681	16104	13271
AAF1613	0	winter	UK	15.75	50.25	NW	42855.28	534.66	57.54	109.77	16.11	1.02	38927	17507	13526
AAF1645	0	winter	UK	15.50	47.00	NW	30908.41	552.33	53.67	53.19	0.33	0.96	31511	17628	12160
AAF1694	0	winter	UK	7.75	45.00	NW	28215.90	563.59	57.43	87.89	2.64	1.00	36078	17506	13130
AAF1880	0	winter	UK	3.25	45.50	NW	42929.44	556.18	58.30	101.65	16.16	1.03	37945	18045	13384
AAF1881	1	winter	UK	8.75	46.75	NW	32467.15	563.37	62.83	117.71	12.09	1.00	39280	16465	13982
AHE7635	0	winter	UK	4.00	45.75	NW	28319.14	549.78	56.75	93.81	2.35	1.01	37177	17206	13336
AL01758	1	summer	Netherlands	5.65	51.98	SW	30013.65	541.71	55.93	117.31	8.03	1.00	38016	16218	13357
APC6349	1	winter	UK	1.25	46.75	NW	26983.49	540.43	55.35	96.30	2.78	0.99	35551	16408	13306
APC6476	1	winter	UK	-1.75	49.00	NW	30276.37	551.47	56.41	92.31	3.63	0.99	35423	16695	13445
APC6732	0	winter	UK	13.25	46.50	NW	32072.26	554.66	58.87	102.83	5.18	1.02	38189	17116	13117
BB26934	1	summer	Netherlands	6.62	52.23	SW	30415.28	532.73	55.27	119.07	8.85	1.00	37362	16138	13942
BC29195	1	summer	Netherlands	5.94	51.82	SW	32071.08	537.62	55.08	112.69	9.60	1.00	36141	16443	13752
BD05464	1	summer	Netherlands	4.69	51.97	SW	24159.38	421.98	42.77	110.21	5.45	1.00	35745	16304	14067
BE81584	1	summer	Netherlands	5.01	52.13	SW	34380.24	557.42	60.61	116.84	12.83	1.03	39600	16478	13349
BE81716	1	summer	Netherlands	5.01	52.13	SW	33146.96	532.76	54.45	112.22	11.22	1.00	36745	16561	13720
BE81799	1	summer	Netherlands	5.01	52.13	SW	36260.02	69.21	7.19	117.47	11.20	1.00	36807	16535	11309
BF05895	1	summer	Netherlands	5.01	52.13	SW	29634.60	526.01	54.49	113.10	8.07	1.00	35972	16412	13898
BF05979	1	summer	Netherlands	5.01	52.13	SW	32999.88	556.19	57.57	117.41	10.45	1.00	37834	16269	13630
BF610	1	summer	Netherlands	6.09	52.29	SW	36471.89	563.51	59.47	116.19	13.42	1.00	39964	16708	12991
BG04063	1	summer	Netherlands	5.01	52.13	SW	40736.48	575.22	61.88	103.87	17.24	1.00	36950	17148	13686
BG04957	1	summer	Netherlands	5.01	52.13	SW	49589.41	543.96	60.00	120.06	13.32	1.01	41191	16934	13140
BH41441	1	summer	Netherlands	6.09	52.29	SW	31028.98	545.91	55.34	99.75	10.03	1.00	36615	17107	13770
BH41477	1	summer	Netherlands	6.09	52.29	SW	38166.11	552.67	58.84	108.98	16.73	1.00	39417	16855	13240
BH60258	1	summer	Netherlands	5.65	51.98	SW	35945.76	551.11	57.94	109.46	14.83	1.00	38361	16956	13634
K505982	0	winter	UK	12.00	47.75	NW	25064.42	541.14	53.87	101.11	5.64	1.00	35515	17152	13349
K505991	1	winter	UK	4.50	46.25	NW	34540.27	537.00	55.99	107.96	14.00	1.00	39297	16913	13411
N268497	1	winter	UK	3.25	46.75	NW	46677.33	538.08	58.45	132.58	17.90	1.01	41364	16181	13539
S523031	1	winter	UK	6.75	46.50	NW	37839.62	523.69	53.35	118.70	13.05	1.01	38930	16353	13520

S609641	1	winter	UK	21.75	50.00	NW	54206.43	614.66	66.07	107.03	13.13	1.00	39008	16910	13459
T012706	1	summer	Austria	13.27	47.71	SW	42364.35	532.25	55.19	124.83	18.50	1.00	37148	15517	13605
T012708	1	summer	Austria	13.22	47.77	S	30902.66	534.87	52.20	69.20	1.56	0.94	32476	17782	13260
T012721	1	summer	Austria	13.95	47.81	S	30149.45	441.02	44.41	108.67	9.34	1.00	37758	16534	13758
T012724	1	summer	Austria	13.95	47.74	SW	30161.28	535.55	52.42	99.60	8.82	1.00	36592	17046	13364
T012725	1	summer	Austria	13.95	47.74	S	25804.28	539.54	52.47	104.43	10.35	1.00	36211	17020	13189
T012731	1	summer	Austria	13.95	47.74	S	29551.62	536.67	54.23	114.21	13.07	1.01	38191	16414	13624
T012732	1	summer	Austria	13.95	47.74	SE	32291.63	533.86	53.13	111.45	9.67	1.00	37621	16762	13729
T012741	1	summer	Austria	14.58	48.20	S	36634.63	534.57	59.19	142.21	13.87	1.00	39192	15237	13475
T012781	1	summer	Austria	16.38	48.25	S	38387.37	553.19	55.78	100.16	16.59	1.00	37307	17118	13529
T012787	1	summer	Austria	13.97	48.64	S	35678.64	620.59	62.20	114.38	13.82	1.00	38087	16587	13041
T012792	1	summer	Austria	13.97	48.64	S	25605.62	534.44	54.33	117.39	6.05	1.00	37345	15821	13634
T012799	1	summer	Austria	13.98	48.64	SE	30184.96	566.16	55.58	117.82	13.44	1.00	37141	16398	13679
T012801	1	summer	Austria	13.97	48.64	S	24133.29	543.64	51.27	92.96	6.17	0.99	35284	17606	13560
T012811	1	summer	Austria	13.98	48.64	SW	29920.06	570.39	55.80	110.24	9.16	1.00	37209	16590	13727
T012816	1	summer	Austria	14.50	48.43	S	23321.57	602.08	56.53	110.15	7.81	0.99	34818	16396	14179
T012825	1	summer	Austria	9.67	47.49	SW	44988.65	608.51	60.68	120.00	15.43	1.00	38445	16197	13316
T012832	1	summer	Austria	9.70	47.45	SW	30687.56	599.49	61.23	111.75	8.24	1.00	36726	16516	13022
T012838	1	summer	Austria	9.70	47.45	SW	42840.90	530.55	55.70	128.95	16.18	1.01	38968	15980	13397
T012847	1	summer	Austria	9.69	47.45	SW	30662.92	536.52	54.32	118.11	10.20	1.00	37829	15935	13746
T012851	1	summer	Austria	9.69	47.45	SW	29040.31	537.01	56.92	120.88	12.49	1.00	39252	16454	13460
T012880	1	summer	Austria	14.17	47.11	SE	28237.94	533.18	53.84	115.72	10.74	1.00	36431	16387	13872
T012889	1	summer	Austria	14.39	47.09	S	36498.50	566.90	58.39	117.08	14.05	1.01	39681	16089	13466
T012928	1	summer	Austria	13.93	46.61	SE	31721.27	537.64	53.96	111.29	12.30	1.00	37062	16428	13465
T012947	1	summer	Austria	14.45	46.63	SE	32125.67	530.44	52.86	105.57	11.22	1.01	37309	16858	13368
T012956	1	summer	Austria	14.45	46.66	S	27530.94	560.48	54.30	75.92	6.85	0.99	33267	19372	13372
T013432	1	summer	Austria	13.98	48.63	S	20414.84	545.99	56.24	83.93	4.26	0.99	11538	5262	10864
T013446	1	summer	Austria	13.97	48.63	S	10062.12	273.64	22.59	83.93	4.26	0.99	34538	17931	13405
T013453	1	summer	Austria	13.97	48.64	SW	24086.44	526.65	49.42	137.57	14.86	1.02	42544	16132	13124
T013459	1	summer	Austria	13.98	48.64	NW	39454.18	719.43	74.46	96.11	10.20	1.00	35994	17223	13509
T013471	1	summer	Austria	13.98	48.63	S	27690.20	523.01	50.79	96.89	11.51	0.99	34601	17065	13650
T013474	1	summer	Austria	13.95	48.65	SW	32740.31	551.32	55.13	96.06	0.92	0.98	33225	14808	13724
T013477	1	summer	Austria	13.98	48.64	S	20996.01	560.08	54.30	86.37	0.45	0.98	32844	14704	13788
T013489	1	summer	Austria	13.87	48.70	SW	17039.32	509.04	45.54	62.75	0.79	0.95	26716	17834	13643
T013494	1	summer	Austria	13.94	48.65	SE	47277.98	439.29	44.81	114.01	18.48	1.02	37543	16501	13633
T013497	1	summer	Austria	13.94	48.66	S	41447.82	547.31	53.06	101.91	13.25	1.00	37317	17299	13454

T013595	1	summer	Austria	13.95	47.82	S	30310.54	537.15	49.20	77.40	13.28	0.99	34723	19017	13275
T013655	1	summer	Austria	14.46	46.63	S	26612.49	530.86	54.82	121.61	7.98	1.01	38098	15825	13224
T013662	1	summer	Austria	14.45	46.63	SE	23426.44	536.36	54.13	105.34	6.09	1.00	36174	16531	13470
T016108	1	summer	Austria	14.51	48.42	SE	35698.38	574.11	57.73	110.32	12.95	1.00	37388	16691	13240
T016112	1	summer	Austria	14.50	48.43	SE	28618.27	528.43	50.74	91.21	10.27	0.99	35147	17449	13373
T016115	1	summer	Austria	14.51	48.42	SE	28178.87	511.21	51.75	100.10	9.44	0.99	32335	16483	13670
T016121	1	summer	Austria	14.50	48.43	S	31595.92	584.29	57.21	114.50	11.22	1.00	37813	16588	13920
T016150	1	summer	Austria	16.39	48.25	SE	25279.48	544.58	50.27	65.45	1.15	0.98	34603	19223	13233
T016153	1	summer	Austria	16.38	48.25	SE	30457.49	556.75	55.00	96.43	9.20	1.00	36927	17604	13692
V771167	0	winter	UK	1.50	41.00	NW	34888.23	543.04	56.36	114.57	17.16	1.02	39452	16954	14085
V771313	1	winter	UK	3.00	46.00	NW	32865.60	535.73	53.25	118.69	6.69	1.00	39712	16293	13469
V856194	0	summer	Netherlands	6.06	53.19	SW	36202.48	558.26	61.76	94.14	8.21	1.01	36778	18192	13788
Z526962	0	winter	UK	-0.75	45.75	NW	52395.88	535.28	56.52	98.05	13.14	1.02	38828	17943	13708
Z629862	1	winter	UK	3.50	48.75	NW	43870.06	565.96	59.57	109.93	16.22	1.00	39113	17065	13217
